# Numerical Modeling of Groundwater Pollution by Chlorpyrifos, Bromacil and Terbuthylazine. Application to the Buñol-Cheste Aquifer (Spain)

**DOI:** 10.3390/ijerph18073511

**Published:** 2021-03-28

**Authors:** Ricardo Pérez-Indoval, Javier Rodrigo-Ilarri, Eduardo Cassiraga, María-Elena Rodrigo-Clavero

**Affiliations:** Instituto de Ingeniería del Agua y Medio Ambiente, Universitat Politècnica de València (IIAMA-UPV), 46022 Valencia, Spain; hidroindoval@gmail.com (R.P.-I.); efc@dihma.upv.es (E.C.); marodcla@upv.es (M.-E.R.-C.)

**Keywords:** pesticides, Chlorpyrifos, Bromacil, Terbuthylazine, numerical modeling, aquifer

## Abstract

Chlorpyrifos, Bromacil and Terbuthylazine are commonly used as insecticides and herbicides to control weeds and prevent non-desirable growth of algae, fungi and bacteria in many agricultural applications. Despite their highly negative effects on human health, environmental modeling of these pesticides in the vadose zone until they reach groundwater is still not being conducted on a regular basis. This work shows results obtained by version 5.08 of the Pesticide Root Zone Model (PRZM5) numerical model to simulate the fate and transport of Chlorpyrifos, Bromacil and Terbuthylazine between 2006 and 2018 inside the Buñol-Cheste aquifer in Spain. The model uses a whole set of parameters to solve a modified version of the mass transport equation considering the combined effect of advection, dispersion and reactive transport processes. The simulation process was designed for a set of twelve scenarios considering four application doses for each pesticide. Results show that the maximum concentration value for every scenario exceeds the current Spanish Maximum Concentration Limit (0.1 μg/L). Numerical simulations were able to reproduce concentration observations over time despite the limited amount of available data.

## 1. Introduction

According to the United States Environmental Protection Agency (USEPA), a pesticide is *“any substance or mixture of substances whose primary purpose is to prevent, destroy, repel, or control a pest”* [1]. Over 500 different pesticide formulations are used in agriculture [2], mainly as herbicides and insecticides, to control weeds and invertebrate pests, and thus improve the crops’ quality [3]. The groundwater of the Júcar River Basin (JRB) in Spain is located under an intense agricultural exploitation area in which the use of pesticides is very frequent [4]. The predominant crops are citrus, although there are also irrigated areas dedicated to vegetables, as well as rainfed areas where cereals, olives and vines are grown [5]. In this context, pesticides used in agriculture reach groundwater mainly by dragging and leaching, thus being able to pollute the aquifers, reducing their quality and posing an environmental risk and even one to human health [3]. Several recent studies at the JRB have detected the presence of pesticides in both surface [6,7,8] and groundwater [9,10,11]. Pesticides in groundwater have been found over the limits established by current legislation due to an excessive use of them and poorly optimized application methods. In fact, it is estimated that only 0.1% of the pesticides that are applied really exert their effect on pathogens, since they are applied preventively. This surplus is subjected to different processes: photodegradation, assimilation by crops, runoff into surface waters, soil adsorption, chemical and/or biological degradation and infiltration into groundwater [12]. Therefore, it is essential to study the dynamics of pesticides in groundwater, both in terms of degradation and transport processes.

In the scientific literature, several models have been developed to predict the transport and fate of pesticides in the environment. Estimations in the vadose zone can be obtained using PESTAN [13], conservative estimates of pesticide concentrations in groundwater are obtained using SCI-GROW [14], general estimations of pesticide fate and transport are obtained using SUTRA [15] and Hydrus [16], the presence of pesticides in surface waters can be analyzed using TOXSWA [17] and pesticide leaching in groundwater can be simulated using PEARL [18].

These mathematical models take into account an important number of physical, chemical and biological processes, as well as pesticide handling practices in the field. These models try to generalize the knowledge of the behavior of pesticides in the analyzed study area, identifying their most important properties, which can be measured in control stations or in laboratories [19]. Moreover, modeling the environmental fate of pesticides has become an important tool for assessing their potential for water contamination [20]. Pesticide models are increasingly used by EU and US authorities to support decisions regarding the approval of pesticide registration.

The use of numerical models has the advantage of being economical and efficient for the evaluation of pesticides while taking into account the large number of relevant aspects of their use in agricultural practices. The selection of the model is justified by the evaluation or the purpose of the study. In many practical situations where not many data are available, using a simple model with fewer parameters is recommended [21]. Numerical modeling to simulating pesticide behavior is an attractive way to perform environmental assessment of agricultural situations [22,23,24,25]. As the first step of the assessment process, it is necessary to identify which pesticide should be used for a specific climate–crop–soil combination, as well as its application rates, so that both crops and the environment are protected.

This work was performed using version 5.08 of the Pesticide Root Zone Model (PRZM5) model [26], included inside the Pesticide Water Calculator (PWC) [27]. PRZM5 is a flexible software that models the fate of pesticides in the environment using relevant local characteristics of climate, soil, hydrology and crop management. It has been developed to estimate pesticide concentrations in groundwater bodies. Output values are obtained in terms of daily, mean and maximum pesticide concentrations, following the regulatory terms established by the USEPA [28].

PRZM5 must be evaluated in terms of the model internal structure, its scientific basics and its capacity to simulate pesticide fate so it can be applied to research.

Therefore, the objective of this study was to use PRZM5 to simulate the fate and transport of three pesticides (Chlorpyrifos, Bromacil and Terbuthylazine) which have been identified inside the Buñol-Cheste aquifer of the Júcar River Basin (JRB). To perform this task, a manual calibration process of the pesticide application was conducted, adjusting the value of the parameter “Amount” of the PRZM5 model, as the exact distribution and applied amount of pesticide mass in the crops were unknown.

To achieve these objectives, it is also necessary to have a detailed and synoptic description of the study area, to have a deep knowledge of the physicochemical characteristics of pesticides and to consider actual values of hydrometeorological, hydrogeological and phenological data. The evaluation of pesticide concentrations is carried out by modeling their behavior on a daily time scale and at a local spatial scale.

Results obtained from this study are important as they provide a more in-depth description of the parameters used in the mathematical modeling of pesticides, while also helping to improve confidence in predictions of pesticide concentrations and facilitating model selection for scientific research and groundwater quality management.

## 2. Materials and Methods

### 2.1. Available Tools for the Numerical Modeling of Pesticide Transport in Soil and Groundwater

Some numerical models are available in scientific literature to approach organic chemicals’ fate and transport in the non-saturated zone (NSZ). Table 1 shows a comprehensive list of numerical models for pesticide transport analysis with their main features [29].

These models allow computation of the characteristics of pesticide transport through the unsaturated zone until it reaches the aquifer. Some models even propose control and correction measures once the soil or groundwater is contaminated. The use of these models allows the prediction of the mobility and persistence of pesticides, establishment of the potential risks that they induce in health or the environment. Most models are based on the previous knowledge about the irrigation management strategies of a certain crop and the use of specific pesticides and fertilizers with which water management is optimized.

In this work it was decided to use the PRZM5 model due to the advantage it offers over other similar numerical models. PRZM5 in the modeling process accounts for:

(i). Data related to climate, soil, hydrology and crop phenology characteristics at local or regional scale.
(ii). Data related to the geometric dimensions and physicochemical characteristics of groundwater bodies.
(iii). Data related to the physicochemical parameters of the pesticides to be evaluated (e.g., vapor pressure, solubility in water and molecular weight).
(iv). Data related to the contaminant fate and transport characteristics (e.g., photolysis, half-life of the pesticide in the soil, foliar degradation and hydrolysis). The model allows selection of the pesticide application date and the corresponding mass applied to the cultivation fields.
(v). PRZM5 output data are shareable in regulatory terms by USEPA as daily, mean and maximum pesticide concentration.
(vi). Output data of pesticide concentrations in short and long simulation periods.

### 2.2. The Pesticide Root Zone Model (PRZM5)

In this work the Pesticide Water Calculator (PWC) (USEPA, Washington, DC, USA) [27] was used. PWC is a graphical user interface to interact both with PRZM5 and the Variable Volume Water Model (VVWM). While PRZM5 focuses on the pesticide transport in the unsaturated zone of the aquifer, the VVWM allows modeling of the movement and degradation of pesticides reaching a surface water body. The research described below was performed using PRZM5. The model has been developed to simulate the fate and transport of pesticides in the unsaturated zone of the aquifer on a daily scale and in one vertical dimension [26]. The main output of the model is the pesticide concentration in the first meter of the saturated zone of a user-set constant depth unconfined aquifer, just below the phreatic surface [28]. The model integrates the essential physical, chemical, and biological processes that occur during pesticide filtration with the vertical movement of water through the soil [30,31,32]. The results shown were obtained using PRZM5, which considers the following processes [26]:

- Crop Growth
- Irrigation
- Precipitation and Snowmelt
- Runoff
- Canopy Water Interception
- Evaporation
- Leaching
- Erosion
- Soil Temperature
- Chemical Application and Foliar Washoff
- Chemical Runoff and Vertical Transport in Soil
- Chemical Volatilization

The mathematical formulations of the different hydraulic and chemical processes that are taken into account by PRZM5 are described below. Water flow in the unsaturated zone is described by Richards [33] as shown in Equation (1):

(1) $$\frac{\partial \theta }{\partial t}=\frac{\partial }{\partial z}\left[K\left(\theta \right)\frac{\partial h}{\partial t}\right]$$

where:

K(θ): unsaturated hydraulic conductivity (cm/s)

θ: soil water content

h: hydraulic head (m)

z: vertical coordinate (m)

t: time (s)

The mass balance equations that account for the different physical–chemical processes suffered by the pesticide through the unsaturated zone are written accounting for the different phases (dissolved, adsorbed and gas) as shown in Equations (2)–(4) [34]:

(2) $$A\Delta z\frac{\partial \left(C_{w}\theta \right)}{\partial t}=J_{D}-J_{V}-J_{\mathrm{DW}}-J_{U}+J_{\mathrm{QR}}+J_{\mathrm{APP}}+J_{\mathrm{FOF}}\pm J_{\mathrm{TRN}}$$

(3) $$A\Delta z\frac{\partial \left(C_{s}\rho_{s}\right)}{\partial t}=-J_{\mathrm{DS}}-J_{\mathrm{ER}}$$

(4) $$A\Delta z\frac{\partial \left(C_{g}a\right)}{\partial t}=-J_{\mathrm{GD}}-J_{\mathrm{DG}}$$

where:

A: transversal section of the soil column (cm^2^)

Δz: depth (cm)

$C_{w}$: concentration of contaminant dissolved in water (g/cm^3^)

$C_{s}$: concentration of contaminant in soil (g/g)

$C_{g}:$ concentration of contaminant in gas phase (g/cm^3^)

θ: volumetric water content (volume of pore water/total volume of the sample) (cm^3^/cm^3^)

a: volumetric air content in soil (cm^3^/cm^3^)

$\rho_{s}$: soil density (g/cm^3^)

t: time (days)

J_D_: mass flux due to dispersion and diffusion in the dissolved phase (g/day)

J_V_: mass flux due to advection in the dissolved phase (g/day)

J_GD_: mass flux due to dispersion and diffusion in the gas phase (g/day)

J_DW_: mass flux due to degradation in dissolved phase (g/day)

J_DG_: mass flux due to degradation in the gas phase (g/day)

J_U_: mass flux from the dissolved phase due to root uptake (g/day)

J_QR_: mass flux from runoff (g/day)

J_APP_: mass flux from pesticide application to soil (g/day)

J_FOF_: mass flux given from the crops to the soil (g/day)

J_DS_: mass flux due to the chemical degradation of adsorbed contaminant (g/day)

J_ER_: mass flux (loss) by dissolution or sediments erosion (g/day)

J_TRN_: mass flux due to other reactions (g/day)

In this newer version of the PRZM5 model, runoff estimation is calculated using the “curve number method” of the National Resources Conservation Service (NRCS) [35]. Runoff calculations start after a minimum amount of precipitation is observed. As precipitation increases, runoff volumes approach precipitation volumes. This simple and efficient runoff estimation method is appropriate for pesticide simulations. PRZM5 uses the Modified Universal Soil Loss Equation (MUSLE) to estimate erosion effects [36].

Water balances are carried out considering runoff, evapotranspiration, irrigation and precipitation processes. The model requires as inputs values of precipitation, temperature, wind speed and evaporation coefficient on a daily scale. The vertical movement of water always develops downwards until the maximum capacity of the soil layers is reached.

Dissolved, adsorbed and gas phase concentrations of the pesticide in the soil are calculated considering the processes of surface runoff, soil erosion, degradation, volatilization, leaf surface washing, plants absorption, filtration, dispersion and sorption. The vertical transport equation is solved using a finite difference scheme [26].

PRZM5 requires the calibration of a set of parameters that describe the chemical characteristics of the pesticide, the pesticide application patterns, the weather characteristics, the soil properties, the irrigation method and those parameters related to crop phenology.

The values of the chemical parameters of the three pesticides that were studied (Chlorpyrifos, Bromacil and Terbuthylazine) were obtained from the existing scientific literature and are summarized in Table 2. Three databases were used: (i) the Pesticide Properties Database (PPDB) [37,38], (ii) PubChem [39] and (iii) the EU Pesticides database [40].

## 3. Case Study. The Buñol-Cheste Aquifer in Valencia Region (Spain)

### 3.1. Description of the Study Area. Hydrogeological Context

**Figure 1:**
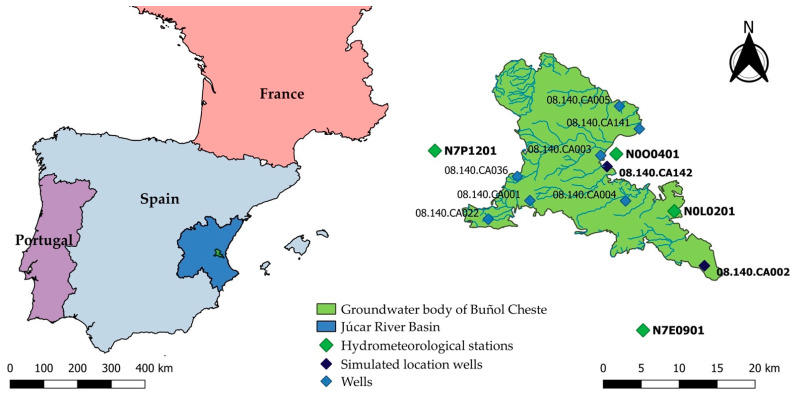
Location of pesticide observation wells inside the Buñol-Cheste Aquifer.

Figure 1 shows the location of the study area, the aquifer of Buñol-Cheste, located on the eastern side of the JRB inside the Valencia Region (Spain). In summer, the average monthly temperature is 22 °C while in winter it is only 6 °C. Average annual precipitation is 350 mm, varying from 280 mm in the southern part of the aquifer to 550 mm in the northern part. In the driest years, the average rainfall is 150 mm/year, while in the wetter years it can reach up to 750 mm/year.

The Buñol-Cheste aquifer has a total area of 542 km^2^ and is characterized by fertile soils and Mediterranean climate that favor agricultural activities. Intense agricultural activity has caused high levels of pesticides to be detected both in ground and surface waters. Furthermore, industrial activity in recent decades has increased due to the decentralization of the industry near the metropolitan area of Valencia City and the improvement of the main communication routes.

The groundwater quality monitoring network is formed by nine observation wells: 08.140.CA001 (Molino Viejo), 08.140.CA002 (La Purísima), 08.140.CA003 (San Álvaro), 08.140.CA004 (Xils), 08.140.CA005 (Calvari), 08.140.CA022 (Barranco Tonau), 08.140.CA036 (La Contienda), 08.140.CA141 and 08.140.CA142 (Llano de Cuarte), located as shown in Figure 1.

**Figure 2:**
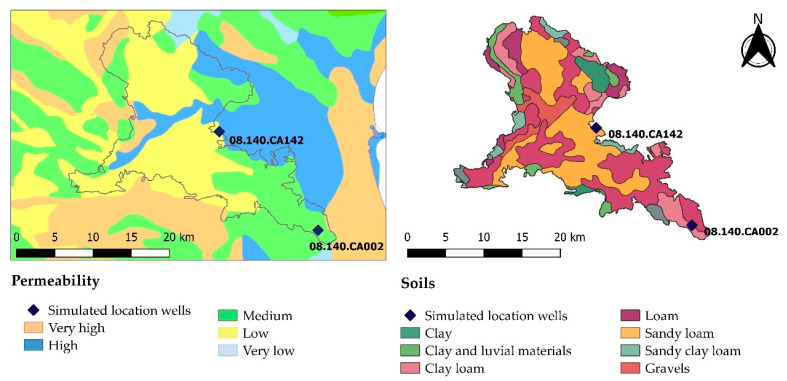
Permeability map (**left**) and lithological map (**right**) of the Buñol-Cheste area.

The Buñol-Cheste geological region is located in the confluence area formed by the foothills of the Iberian and the Betic Mountain Ranges in Eastern Spain. Therefore, groundwater flows mainly from west to east. According to the information obtained from the current JRB hydrological planning [41], the Buñol-Cheste aquifer can be divided into five zones with similar hydrogeological properties, in which saturated hydraulic conductivity (K) varies as shown in Figure 2.

The aquifer is formed by alternating higher-K materials (mainly gravel and sand) and lower-K materials (silt and clay) spatially distributed as shown in Figure 2. Table 3 summarizes the available geological information inside the Buñol-Cheste aquifer for every geological unit.

Table 3 shows that the Buñol-Cheste aquifer presents an important geological complexity with a great variety of structures and geological features that greatly affect its hydrogeological behavior. The complexity of the geological units is the reason why the hydrogeological functioning is not fully known. The main materials capable of storing and transmitting water in the area are carbonates. In almost all cases the aquifer can be considered to be under unconfined conditions. Only in some observation wells the aquifer shows semi-confined behavior. According to the lithostratigraphic column there are a series of carbonate formations capable of developing aquifers. Table 4 shows the details of the available hydrogeological properties of each aquifer section.

### 3.2. Available Pesticide Observations from the Monitoring Network

Pesticides have been found in high concentrations in the different groundwater bodies that form the JRB. Following current Spanish regulation (Royal Decree 1514/2009), a groundwater body is considered contaminated when a pesticide or by-product exceeds a Maximum Concentration Level (MCL) equal to 0.1 μg/L or when the total concentration of all these compounds is greater than 1 μg/L [42]. According to this criterion, in the Buñol-Cheste Aquifer, non-compliances due to Bromacil, Chlorpyrifos and Terbuthylazine have been observed. The use of Bromacil and Chlorpyrifos has been restricted or prohibited for years, so it is to be expected that their current concentrations in groundwater will decrease with time. However, pesticides are still detected in concentrations lower than 0.1 mg/L, which still makes it necessary to maintain strict control of these chemicals [5].

**Figure 3:**
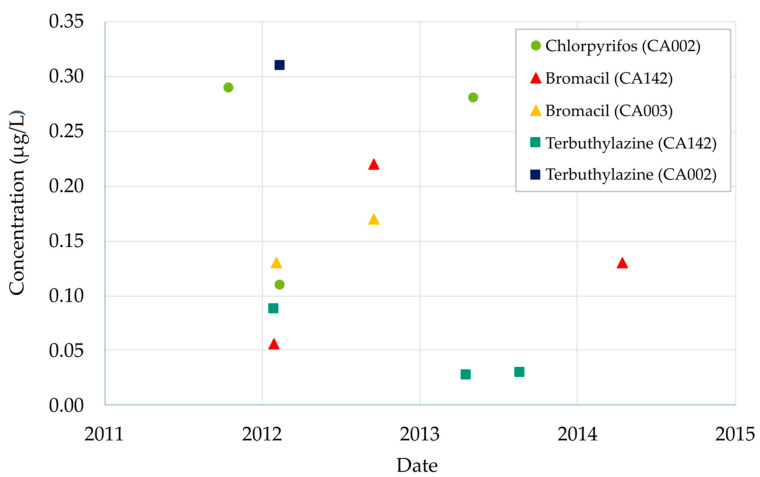
Temporal evolution of Chlorpyrifos, Bromacil and Terbuthylazine concentrations in the Buñol-Cheste Aquifer from 2011 to 2014.

Figure 3 shows the evolution over time of the registered pesticide concentrations in the aquifer between 2011 and 2014. MCLs were exceeded eight times in the three observation wells (CA002, CA003 and CA142) during the 2011–2015 period.

Chlorpyrifos concentrations have exceeded the MCL three times (November 2011, March 2012 and June 2013) in observation well 08.140.CA002 (La Purísima). Concentrations in these wells were 0.29, 0.11 and 0.28 μg/L, respectively.

Bromacil was detected three times in observation well 08.140.CA142 (Llano de Cuarte): March 2012 (0.06 μg/L), October 2012 (0.22 μg/L) and April 2014 (0.13 μg/L). Furthermore, Bromacil concentrations were also found in well 08.140.CA003 (San Álvaro) on the same dates. This spatial–temporal coincidence may be due to the fact that Bromacil was used on one-single common application. Bromacil is a product that has been banned for years and its presence may be due to sporadic use, without discarding possible analytical inaccuracies.

Terbuthylazine’s concentrations almost reached the MCL in well 08.140.CA142 (Llano de Cuarte) in March 2012 (0.09 μg/L) and May and October 2013 (0.03 μg/L in both dates). There is another record of Terbuthylazine in well 08.140.CA002 (La Purísima) in 2012 (0.31 μg/L). However, this record was not confirmed by subsequent measurements.

In order to explain the causes of non-compliance by Terbuthylazine in the groundwater mass, two key points must be addressed. The first one is the location of the monitoring wells. Although most of the surface of the aquifer is covered by forest lands, the observation wells are located in agricultural areas which must be the source of contamination. Despite Terbuthylazine currently not being used by farmers, it was a basic herbicide in citrus cultivation until recent years and is probably still being used. The second aspect to address refers to the physicochemical characteristics of the pesticides. In this work, the Groundwater Ubiquity Score (GUS) [43] was used as a tool to classify pesticides depending on their risk of leaching into groundwater. Terbuthylazine shows a high GUS index (3.07) which indicates that the pesticide actually reaches the aquifer saturated zone.

Chlorpyrifos leaching potential is medium–high (GUS index = 2.57), so its presence in the aquifer is not surprising. However, the fact that it is detected in a single point repeatedly in time suggests the existence of a very localized use.

The other non-compliance situation detected in this aquifer corresponds to Bromacil, which is a highly soluble herbicide, with high mobility (GUS index = 3.44) and moderate persistence. Bromacil was detected frequently and in concentrations of the order of up to 0.2 μg/L in well 08.140.CA142 (Llano de Cuarte). It is striking that the use of this compound in citrus cultivation has been prohibited since 2002, although the last authorizations for its use were given in 2007. The fact that such high concentrations appear between 2012 and 2014 suggests that, despite its prohibition, it has still been used in some sectors. An alternative explanation may be related to the fact that Bromacil is highly soluble, and it shows high persistence. In such a case, its presence should tend to disappear with time.

Therefore, pesticide observations from the monitoring network justify the need for controlling the presence of pesticides in the aquifer, especially in wells 08.140.CA142 and 08.140.CA002 in order to analyze their evolution with time.

### 3.3. Data Sources

Data used in this research were obtained from different sources:

(i). Records of the Valencia Water Regional Authority (JRB);
(ii). JRB’s Automatic Hydrographic Information System (SAIH);
(iii). The Spanish National Meteorological Agency (AEMET);
(iv). The Atlas of Solar Radiation in Spain using climate data from Satellite Application Facilities (SAF EUMETSAT);
(v). Data and cartography provided by JRB.

The climatic variables studied were maximum temperature (T_max_, °C), minimum temperature (T_min_, °C), wind speed (v, m/s), relative humidity (RH, %) and precipitation (PP, mm/d).

Table 5 shows the application patterns for each pesticide. The number and dates of the applications were obtained from surveys of farmers [44]. The results of these surveys do not provide information about the exact pesticide doses applied so their distribution is not known with certainty. Therefore, as only ranges of pesticide applications are known, their final value and its distributions were calibrated by the modeler after an iterative process that allowed the establishment the value of the PRZM5 parameter “Amount” for each one of the three pesticides. These amounts of pesticide which are provided annually on the area associated with every observation well are shown in Table 5.

Table 6 shows the information sources from which the weather data were obtained for every pesticide. The location of these hydrometeorological stations is shown in Figure 1.

The Evaporation Factor was computed following Meyer’s formula in Equation (5) [46]:

(5) $$E=C\left(e_{a}-e\right)\left(1+\frac{v}{16}\right)$$

where:

E: daily evaporation (mm/d);

C: empirical coefficient taken as 0.36;

e_a_: saturation vapor pressure at the water surface (mm hg);

e: air vapor pressure (mm hg);

v: wind speed (km/h) measured at 7.64 m above the surface.

When dealing with citrus crops, the type of irrigation is under canopy. The maximum amount of supplied water is 72 cm/day, and the Soil Irrigation Depth is equal to the full depth of the Root Zone. The unsaturated zone was vertically discretized in seven horizons for the simulation of Chlorpyrifos in well 08.140.CA002 (La Purísima) and also for the simulation of Bromacil and Terbuthylazine in well 08.140.CA142 (Llano de Cuarte). Table 7 and Table 8 show the soil properties for each one of these wells. Soil data included in all the simulations were obtained from the Hydrological Plan of the Júcar River Basin District 2015–2021 [4].

Variation in soil temperature affects pesticide degradation. To simulate this effect, it is necessary to calibrate the values of Lower Boundary Condition Temperature and the Albedo. The values corresponding to these parameters after a manual calibration process are 12 °C and 0.2, respectively. In this work a single annual crop cycle is assumed. The emergence, maturity, and harvest dates for the simulation of the three pesticides are 1 April, 1 July and 1 October, respectively. Table 9 summarizes the information included in the model regarding the characteristics of the vegetation cover.

For all the three pesticides, it is assumed that the pesticide remains on the leaves after harvest. Afterwards, this residue is mobilized through the unsaturated zone subjected to decay and washing processes.

## 4. Results

The use of the PRZM5 model allowed the simulation of pesticide concentrations in the Buñol-Cheste Aquifer accounting for the available data. Once the model was calibrated, the results obtained from these simulations are shown below for each one of the pesticides under analysis.

### 4.1. Chlorpyrifos Simulations

**Figure 4:**
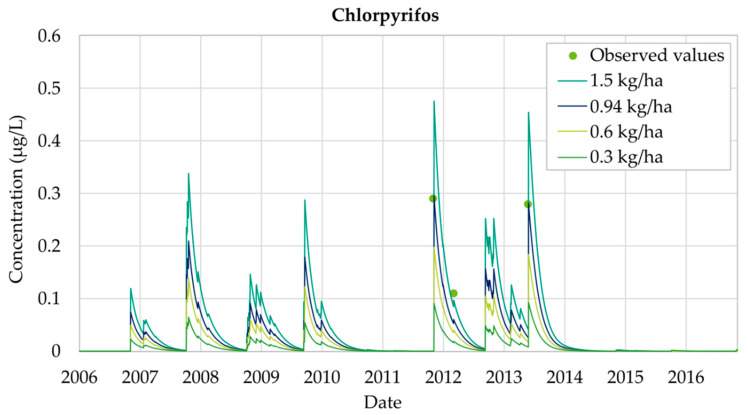
Chlorpyrifos simulations in Well 08.140.CA002 (La Purísima) in 2006–2016.

Figure 4 shows the effect of increasing the application of Chlorpyrifos from 0.3 kg/ha to 1.5 kg/ha. It was observed that the evolution over time of the experimental measurements and the simulated values followed the same pattern. As shown in Figure 4, in the period 2006 to 2010 there is an increase in the concentration of Chlorpyrifos, exceeding the MCL considered by Spanish legislation. The results show that, by the end of 2011 Chlorpyrifos breakthrough curves abruptly increased until they reached maximum values. In 2012, concentrations continued to oscillate around 0.10 μg/L while the application continues. Finally, during the years 2013 and 2014 Chlorpyrifos concentrations decrease down to zero. It was clearly appreciated that, in 2014, a decrease in concentration began. Figure 4 also shows that the model has been properly calibrated as the observed values are reproduced by the simulation results. However, not all observations are adjusted in the same way. Some correspond to local maximum concentration points while others are included inside the general trend of the simulated results.

**Figure 5:**
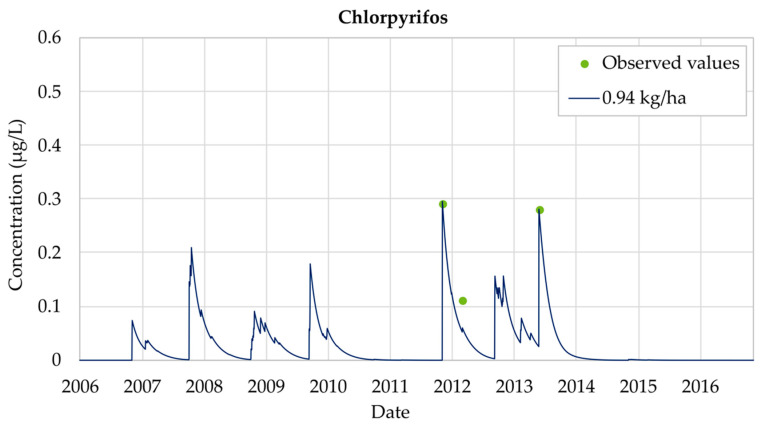
Chlorpyrifos calibrated simulation at Well CA002 (La Purísima) considering an annual application equal to 0.94 kg/ha between 2006 and 2016.

The results obtained for the calibrated model show that an annual application equal to 0.94 kg/ha of Chlorpyrifos is the one that provides the best adjustments to the observed data. Figure 5 shows the evolution of Chlorpyrifos concentrations in the aquifer for these calibrated simulations. Concentration values observed on 22 November 2011 equal to 0.29 μg/L are exactly equal to the simulated results. Furthermore, the second concentration observation, measured on 21 March 2012 is equal to 0.11 μg/L, while the simulated concentration at that time is 0.09 μg/L (18% lower than the observed data). Finally, the third observation record on 17 June 2013 equal to 0.28 μg/L is also adequately reproduced.

Chlorpyrifos analysis shows that the simulated concentrations do not present significant differences with the observed concentrations and accurate results were achieved, maintaining good adjustments throughout the 2006–2016 simulation period. Model results show that general trends are properly reproduced and MCL values (0.1 μg/L) are only temporarily overcome. The use of the model allows confirmation that maximum Chlorpyrifos concentration values coincide with times when applications to the soil occur.

### 4.2. Bromacil Simulations

**Figure 6:**
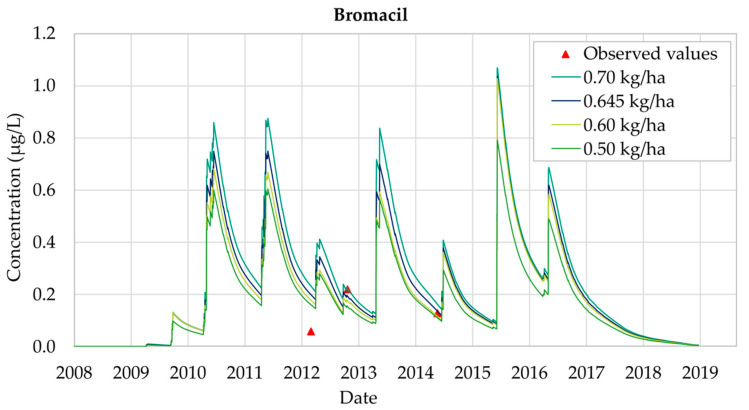
Bromacil simulations in Well CA142 (Llano de Cuarte) in 2008–2018.

Figure 6 shows the temporal evolution of the Bromacil concentration in Well 08.140.CA142 (Llano de Cuarte) for the period 2008–2018. It can be seen that the concentration curves follow a similar pattern for every type of application, since they all show a maximum value and a symmetric distribution.

Four different sets of scenarios were designed, considering different annual Bromacil application values (0.50 kg/ha, 0.60 kg/ha, 0.645 kg/ha and 0.70 kg/ha). Bromacil was detected for the first time on 11 April 2009 and disappeared on 6 August 2018, so its presence in groundwater was equal to 2598 days. It was observed that the simulated concentrations exceed the MCL threshold of 0.1 μg/L, showing a significant prevalence throughout all the simulation periods.

**Figure 7:**
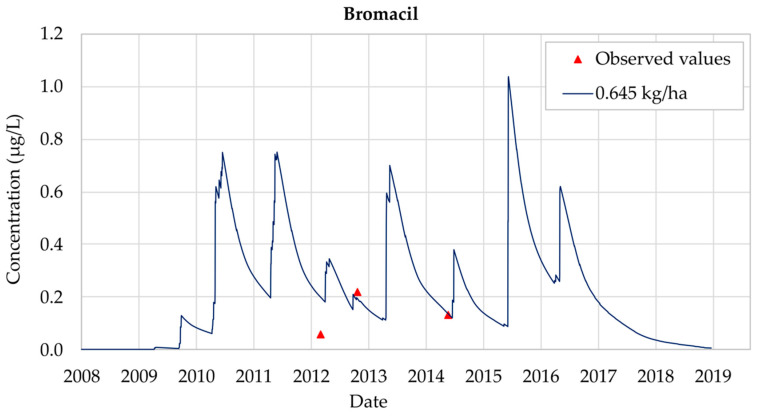
Bromacil calibrated simulation at Well CA142 (Llano de Cuarte) considering an annual application equal to 0.645 kg/ha between 2008 and 2018.

The best calibration of the model was obtained considering an annual Bromacil application equal to 0.645 kg/ha. Figure 7 shows the results obtained for this scenario, for which all the observed values but one are well reproduced. The only observation that is not properly reproduced is the one taken on 3 July 2012. On this date the observed concentration was equal to 0.06 μg/L, while the simulation result overestimates this value (0.20 μg/L). There are many reasons that can cause these differences, including errors in the measurement process. The record on 25 October 2012 is equal to 0.22 μg/L, while the simulated value is 0.20 μg/L. On 28 May 2014, a Bromacil concentration equal to 0.13 μg/L was observed and this value is accurately reproduced by the model. On 15 June 2015 the maximum concentration value was reached, and concentrations finally decreased to zero by the end of 2018.

Bromacil analysis shows that the simulated concentrations do not show significant differences with the observed concentrations, though the results are not so accurate as those obtained for Chlorpyrifos. However, throughout the 2008–2018 simulation period general trends are properly reproduced. The use of the PRZM5 model allows prediction of the maximum Bromacil concentration values, which were not included inside the set of observation values showing that MCLs (0.1 μg/L) are generally overcome throughout the whole simulation period.

### 4.3. Terbuthylazine Simulations

Terbuthylazine simulations were performed at Well 08.140.CA142 (Llano de Cuarte) following the same procedure as explained before for Chlorpyrifos and Bromacil. Despite results for Terbuthylazine not being as accurate as those obtained for Chlorpyrifos and Bromacil, the modeling process provides results of great importance to understand the behavior of Terbuthylazine in the Buñol-Cheste Aquifer.

**Figure 8:**
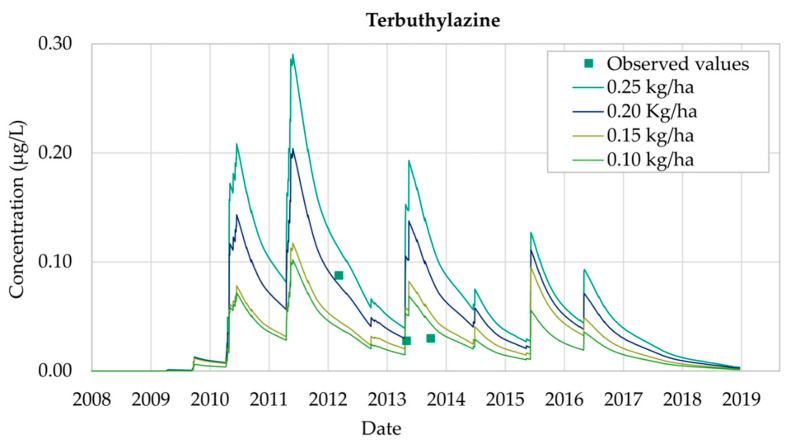
Terbuthylazine simulations in Well 08.140.CA142 (Llano de Cuarte) in 2006–2016.

Figure 8 shows the evolution of Terbuthylazine for a set of four scenarios considering different values of the annual applications of the pesticide (0.25 kg/ha, 0.20 kg/ha, 0.15 kg/ha and 0.10 kg/ha). Model predictions are such that the date on which the maximum values are observed is the same for the four scenarios. Model results show that simulated concentrations are proportional to the value of the application amount, so the peak value of the concentration curve is proportional to the corresponding application value. The simulated Terbuthylazine concentrations are higher than MCL between 2010 and 2014 for two of the scenarios (0.25 kg/ha and 0.20 kg/ha). Terbuthylazine concentrations are under the MCL during the whole simulation period for the other two scenarios (0.15 kg/ha and 0.10 kg/ha), disregarding that the MCL limit is reached once in May 2011 (scenario 0.15 kg/ha). Results for all the scenarios show that, later than 2016, Terbuthylazine concentrations in the well decrease, reaching zero by the end of 2018.

**Figure 9:**
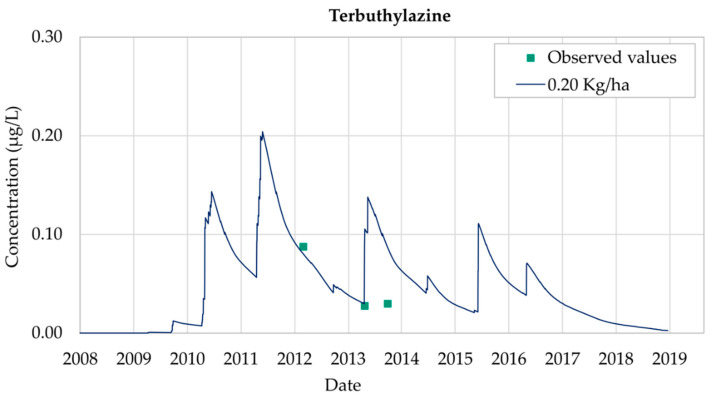
Terbuthylazine calibrated simulation at Well 08.140.CA142 (Llano de Cuarte) considering an annual application equal to 0.20 kg/ha between 2008 and 2018.

The best fit of the observed data is obtained with an application value of 0.20 kg/ha. Figure 9 shows the simulated values of Terbuthylazine concentrations in groundwater for this scenario during the simulation period 2008–2018, after the calibration process had been performed. The maximum simulated concentration value is 0.20 μg/L, obtained on 19 May 2011.

As Terbuthylazine has a high rate of mobilization and persistence, the simulated values oscillate throughout the simulation period in the aquifer.

The concentration of Terbuthylazine registered on 7 March 2012 is equal to 0.09 μg/L while the model simulation was equal to 0.08 μg/L. Despite the model being able to accurately represent the observation taken on 30 May 2013, results are inconsistent with the observation made on 3 October 2013. The calibration process led to the conclusions that simulated concentrations are usually located in time before the observed concentrations.

Terbuthylazine analysis shows that the simulated concentrations are not as accurate as those obtained for Chlorpyrifos and Bromacil. However, general trends are properly reproduced throughout the 2008–2018 simulation period. The use of the model allows prediction of the maximum Terbuthylazine concentration values, which were not included inside the observation values, showing that simulated concentration values are usually under the MCL values (0.1 μg/L) throughout the whole simulation period.

## 5. Discussion

PRZM5 was used to simulate Chlorpyrifos, Bromacil and Terbuthylazine concentrations in groundwater in the Buñol-Cheste aquifer. For each pesticide and for every application pattern, the following variables were computed: (i) Peak concentration value (μg/L); (ii) Average concentration value (μg/L); (iii) Number of days in which the simulated concentration is higher than the MCL (0.10 μg/L); (iv) Date on which the pesticide concentration is reduced to zero, and (v) Number of days in which pesticide concentration in groundwater is greater than zero. Table 10 shows the results summary using these five variables.

A set of twelve scenarios (three pesticides and four application doses) was designed to analyze pesticide concentration in groundwater. The results show that the maximum concentration value for every scenario exceeds the Spanish MCL (0.1 μg/L). As expected, simulations results justify that the simulated peak concentrations increased when the application dose increased. However, some differences were found when analyzing the behavior of each pesticide individually. Average concentrations are lower than the MCLs for Chlorpyrifos and Terbuthylazine but exceed the MCL for Bromacil.

**Figure 10:**
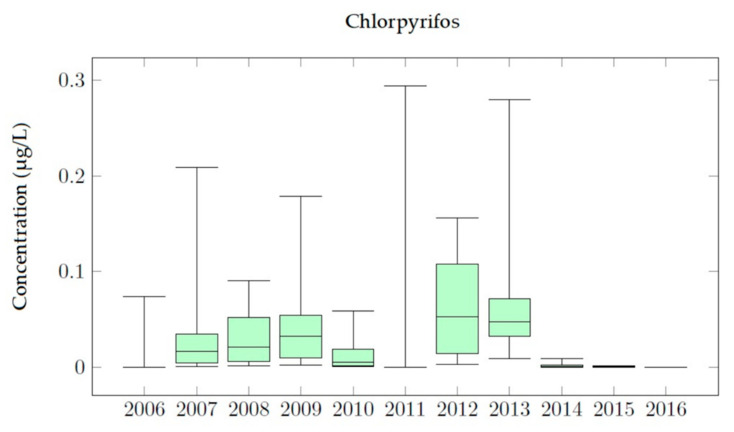
Box plot diagram of Chlorpyrifos simulations in Well 08.140.CA002 (La Purísima) in 2006–2016.

**Figure 11:**
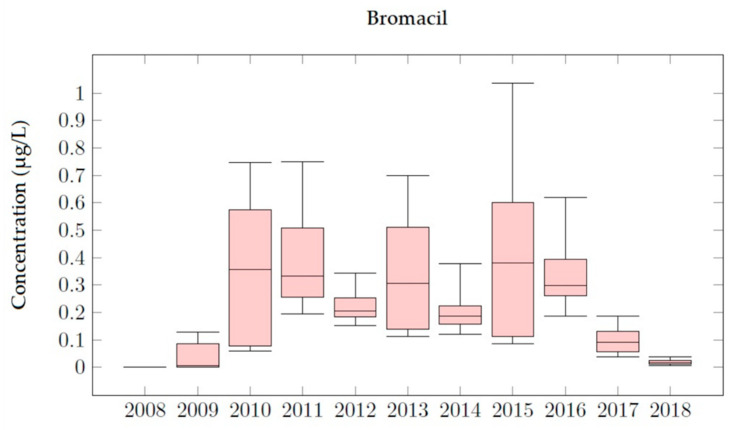
Box plot diagram of Bromacil simulations in Well 08.140.CA142 (Llano de Cuarte) in 2008–2018.

**Figure 12:**
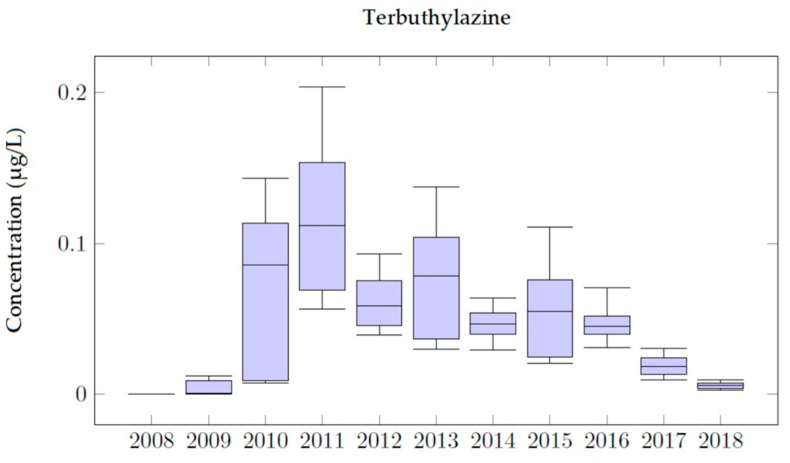
Box plot diagram of Terbuthylazine simulations in Well 08.140.CA142 (Llano de Cuarte) in 2008–2018.

The number of days for which pesticide concentration in groundwater is higher than the MCL are around 2500 days for Bromacil, despite variations in the application dose, meaning that Bromacil is a more persistent compound. For Chlorpyrifos and Terbuthylazine the number of days for which pesticide concentration in groundwater is higher than MCLs change a lot with the application dose (less than 550 days for Chlorpyrifos and less than 1000 days for Terbuthylazine). Terbuthylazine is more sensitive to application dose changes (e.g., doubling the dose increases the permanence of the contaminant 64 times). The persistence of pesticide in the observation wells is similar for Bromacil and Terbuthylazine (between 3000 and 3400 days) and lower than 2700 days for Chlorpyrifos. To analyze the evolution in time of the concentration of the simulated pesticides the corresponding box plot diagrams with the main statistics were obtained (Figure 10, Figure 11 and Figure 12). The main effects that were observed on the JRB area are described below.

In the Chlorpyrifos analysis, it can be seen that in the years 2012 and 2013 the dispersion of the concentration values is greater than in the other years. Furthermore, in these years, the MCL is exceeded. By 2014, the concentrations of Chlorpyrifos had decreased in the Buñol-Cheste Aquifer, while the concentrations of the other two pesticides remained persistent. Bromacil simulations indicate that this pesticide is moderately persistent and highly mobile in the environment. In the 2010–2015 period, the highest records of this pesticide were recorded, reaching values up to 1.0 μg/L. Therefore, Bromacil is the pesticide that most affects the Buñol-Cheste Aquifer. The highest concentration is observed for the year 2015 when 84% of the simulated concentration values are above the MCL. The analysis carried out for Terbuthylazine shows that the concentration values for this pesticide are not as high as those found for Bromacil and Chlorpyrifos, but they are very close to the MCL. Simulated concentration values for Terbuthylazine in the Buñol-Cheste Aquifer do not exceed 0.20 μg/L. During the 2012–2016 period, concentrations varied between 0.10 μg/L in 2013 and 0.02 μg/L in 2015. During the years 2017 and 2018 the concentration values were below 0.05 μg/L.

The simulations carried out with PRZM5 in this study identified that the annual application dose of the pesticide (parameter “Amount” in PRZM5) is the most sensitive parameter when considering the soil and climate conditions of the Júcar River Basin. Therefore, the “Amount” parameter must be carefully calibrated when the exact values of the pesticide applications are not available.

## 6. Conclusions

This work provides a first step towards the use of numerical modeling techniques for the analysis of pesticide behavior in the Buñol-Cheste aquifer (Spain). For the first time, this original research shows results obtained by the PRZM5 model to simulate the fate and transport of Chlorpyrifos, Bromacil and Terbuthylazine between 2006 and 2018. Two different observation wells (Well 08.140.CA002-La Purísima and Well 08.140.CA142-Llano de Cuarte) where pesticide concentrations exceed the current Spanish MCL (0.10 μg/L) were selected as sources of concentration data. A set of twelve different scenarios, considering four application doses for each one of the three pesticides, were analyzed.

One of the main purposes of this study is the generation of information and tools that organize and facilitate the performance of risk assessments of pesticides in the Buñol-Cheste aquifer. The original information that was produced during the development of this work includes:

(i). a set of databases, according to the parameters required by an unsaturated contaminant transport model;
(ii). a sensitivity analysis of the model parameters, including the pesticide annual application dose (kg/ha);
(iii). estimation of the annual concentration values of every pesticide under a risk assessment framework for the first time in the Buñol-Cheste aquifer.

The greatest difficulty encountered when carrying out the simulations was related to the calibration of the model parameters (Curve Number, USLE factors, soil adsorption coefficient, among others). Given the lack of sufficient field data, in this study it was decided to perform a manual calibration of the pesticide application doses, described by the parameter “Amount” in PRZM5, following a trial-and-error process, modifying this parameter individually and analyzing the change of the model results. Once the manual calibration process was performed, it can be seen that the model results reproduce the concentration observations.

Chlorpyrifos analysis leads to accurate results, maintaining good adjustments between observations and simulated results throughout the 2006–2016 simulation period. Model results show that general trends are properly reproduced and MCL values (0.1 μg/L) are only temporarily overcome. The use of the model allows confirmation that maximum Chlorpyrifos concentration values coincide in times when applications to the soil occur.

Bromacil analysis shows that the simulated concentrations do not present significant differences with the observed concentrations, though the results are not as accurate as those obtained for Chlorpyrifos. However, throughout the 2008–2018 simulation period, general trends are properly reproduced. The use of the model allows prediction of the maximum Bromacil concentration values, which were not included inside the observation values, showing that MCLs (0.1 μg/L) are generally overcome throughout the whole simulation period.

Terbuthylazine analysis shows that the simulated concentrations are not as accurate as those obtained for Chlorpyrifos and Bromacil. However, throughout the 2008–2018 simulation period, general trends are properly reproduced. The use of the model allows prediction of the maximum Terbuthylazine concentration values, which were not included inside the observation values showing that simulated concentration values are usually under the MCL values (0.1 μg/L) throughout the whole simulation period.

In conclusion, simulation results indicate that, despite the lack of field information, the PRZM5 model is a valid tool to study the behavior of pesticides in the Buñol-Cheste aquifer as it provides valuable information to stakeholders and environmental authorities.

## Figures and Tables

**Table 1:** Main features of available numerical models for pesticide analysis.

Model	Ref.	Objectives	NSZ *	SZ **	1D–2D–3D	Processes
PESTAN	[13]	Pesticide concentration in soil	yes	no	1D	Advection, dispersion and reactions
PRZM5	[26]	Pesticide concentration in soil and groundwater	yes	yes	1D	Advection, dispersion, reactions and root interactions
SCI-GROW	[14]	Pesticide analysis	no	yes		Under development
SUTRA	[15]	Heat and solute transport	yes	yes	3D	Advection, diffusion and sorption
HYDRUS	[16]	Heat and solute transport	yes	yes	3D	Advection, dispersion and reactions between phases
TOXSWA	[17]	Pesticide in aquatic ecosystems	yes	no	2D	Transformation, sorption, volatilization, advection, dispersion and diffusion
PEARL	[18]	Pesticide leaching in groundwater, infiltration and persistence	yes	yes	1D	Advection, dispersion, sorption, volatilization, transformation, evaporation and plant absorption

* NSZ: Non-Saturated Zone; ** SZ: Saturated Zone.

**Table 2:** Pesticide physicochemical parameters.

Parameter	Unit	Chlorpyrifos	Ref.	Bromacil	Ref.	Terbuthylazine	Ref.
Sorption Coefficient	ml/g	18.15	[38]	230	[39]	250	[40]
Hydrolysis Half Life	days	25.5	[38]	10	[39]	11	[40]
Surface Soil Half Life	days	21	[38]	21	[39]	30	[40]
Soil Reference Temperature	°C	20	[40]	20	[40]	20	[40]
Molecular Weight	g/mol	350	[37]	261.12	[37]	229.71	[37]
Vapor Pressure	torr	0.00013	[37]	0.00013	[37]	0.15	[37]
Solubility	mg/L	2	[37]	700	[37]	8.5	[37]
Henry’s Constant	--	0.000164	[39]	0.000000037	[39]	0.00405	[39]
Air Diffusion Coefficient	cm^2^/day	4300	[39]	4300	[39]	4300	[39]
Henry’s Heat	J/mol	83,860	[39]	83,860	[39]	83,860	[39]

**Table 3:** Geological units inside the Buñol-Cheste aquifer [41].

Name	Lithology	Thickness (m)	Age	Hydrogeological Condition
Buntsandstein	Sandstone and conglomerates	170–300	Lower Triassic	Low-K
Muschelkalk	Dolomites, limestones and marls	85–130	Middle Triassic	Medium-K
Keuper	Plasters and clays	40–100	Upper Triassic	Impermeable
Jurassic–Cretaceous	Limestones and dolomites	1000–1200	Jurassic–Cretaceous	Variable K
Lower Miocene	Conglomerates, sands and limestones	200	Lower Miocene	Variable K
Upper Miocene	Lake limestones		Upper Miocene	Aquifer
Quaternary	Gravels, sands, silts and clays		Pleistocene–Holocene	High-K

**Table 4:** Hydrogeological properties of the Buñol-Cheste aquifer subsections [41].

Hydrogeological Section	Nature	Thickness (m)	Hydrostatic Conditions	Permeability
Yátova Quaternary	Carbonated detrital		Unconfined	
Southern Miocene	Carbonated	200–600	Confined	
Rambla de Bugarra Plioquaternary	Carbonated detrital		Mixed	
Godelleta Miocene	Carbonated		Unconfined	Medium-K
Cheste Plioquaternary	Carbonated detrital	90 (minimum)	Unconfined	
Buñol-Cheste Jurassic–Tertiary	Carbonated detrital		Mixed	High-K
Chiva-Cheste Plioquaternary	Carbonated detrital		Mixed	
Cañada Fría Jurassic	Carbonated detrital		Mixed	High-K
Urrea-Pedrizos Miocene	Carbonated detrital	78–267	Unconfined	Karst
La Balsica Upper Cretaceous	Carbonated detrital		Mixed	
Lomayma Jurassic	Carbonated detrital		Mixed	
El Palmeral Upper Cretaceous	Carbonated detrital	350 (minimum)		
Serretilla Jurassic	Carbonated	700 (maximum)	Mixed	Karst

**Table 5:** Pesticide application patterns.

Parameter	Unit	Chlorpyrifos		Bromacil		Terbuthylazine	
Amount (total)	kg/ha	0.94		0.635		0.20	
Application Method	–	Above Crop		Above Crop		Above Crop	
Number of Applications	–	5		3		2	
		Date	kg/ha	Date	kg/ha	Date	kg/ha
		1 April 1 May 1 June 1 July 1 August	0.40 0.15 0.03 0.26 0.10	1 April 1 May 1 June	0.245 0.19 0.20	10 April 10 May	0.10 0.10

**Table 6:** Weather data information sources.

		Hydrometeorological Station				
Well	Pesticide	Precipitation cm/d	Temperature °C	Wind Velocity cm/d	Evaporation Factor cm/s	Solar Radiation La/d
080.140.CA002 La Purisima	Chlorpyrifos	N0L0201	N7E0901	N7E0901	Meyer’s formula	Solar radiation Atlas in Spain [45]
08.140.CA142 Llano de Cuarte	Bromacil Terbuthylazine	N0O0401	N7P1201	N7P1201		

**Table 7:** Soil column parameters. Well 08.140.CA002 (La Purísima).

Parameter	Unit	Well 08.140.CA002 (La Purísima)						
Layer		1	2	3	4	5	6	7
Thickness	cm	10	10	20	20	20	20	400
Density	g/cm^3^	1.2	1.2	1.2	1.3	1.35	1.35	1.48
Max. Cap.	-	0.207	0.207	0.207	0.207	0.270	0.270	0.270
Min. Cap.	-	0.095	0.095	0.095	0.095	0.117	0.117	0.117
Organic Carbon	%	1.7	1.7	0.7	0.7	0.3	0.3	0.3
Nitrogen	-	1	1	1	1	1	1	1
Sand	%	48	48	58	53	40	40	40
Clay	%	16	16	16	16	28	28	28

**Table 8:** Soil column parameters. Well 08.140.CA142 (Llano de Cuarte).

Parameter	Unit	Well 08.140.CA142 (Llano de Cuarte)						
Layer		1	2	3	4	5	6	7
Thickness	cm	10	10	20	20	20	20	500
Density	g/cm^3^	1.3	1.25	1.25	1.25	1.25	1.25	1.49
Max. Cap.	-	0.318	0.339	0.339	0.339	0.339	0.339	0.207
Min. Cap.	-	0.197	0.239	0.239	0.239	0.239	0.239	0.095
Organic Carbon	%	0.26	0.12	0.12	0.12	0.12	0.12	0.71
Nitrogen	-	10	1	1	1	1	1	10
Sand	%	40	35	35	35	35	35	80
Clay	%	60	65	65	65	65	65	20

**Table 9:** Vegetation cover characteristics.

Parameter	Unit	Value	Source
Root depth	cm	50	Survey
Canopy cover	%	80	Survey
Canopy height	cm	200	Survey
Canopy holdup	cm	0.15	Survey

**Table 10:** Results summary. Pesticide concentration characteristics for every application dose.

	**Chlorpyrifos**			
**Application Dose**	**0.3 kg/ha**	**0.6 kg/ha**	**0.94 kg/ha**	**1.5 kg/ha**
Peak concentration (μg/L)	0.0920	0.1976	0.2946	0.4750
Average concentration (μg/L)	0.0081	0.0173	0.0258	0.0415
Days > 0.10 μg/L	0	124	301	538
C = 0 date	30 November 2013	13 January 2014	11 February 2014	18 March 2014
Duration (days)	2580	2624	2653	2688
	**Bromacil**			
**Application dose**	**0.50kg/ha**	**0.60 kg/ha**	**0.645 kg/ha**	**0.70 kg/ha**
Peak concentration (μg/L)	0.7920	1.0289	1.0365	1.0696
Average concentration (μg/L)	0.1720	0.1995	0.2169	0.2442
Days > 0.10 μg/L	2342	2573	2598	2656
C = 0 date	10 June 2018	24 July 2018	6 August 2018	28 August 2018
Duration (days)	3347	3391	3404	3426
	**Terbuthylazine**			
**Application dose**	**0.10 kg/ha**	**0.15 kg/ha**	**0.20 kg/ha**	**0.25 kg/ha**
Peak concentration (μg/L)	0.1020	0.1173	0.2093	0.2905
Average concentration (μg/L)	0.0229	0.0292	0.0458	0.0625
Days > 0.10 μg/L	8	58	512	907
C = 0 date	20 December 2017	16 April 2018	15 August 2018	18 October 2018
Duration (days)	3010	3127	3248	3312

## Data Availability

Data used in this research were obtained from different sources: (i) Records of the Valencia Water Regional Authority (JRB). Restrictions apply to the availability of these data. Data was obtained JRB and are available from the authors with the permission of JRB; (ii) JRB’s Automatic Hydrographic Information System (SAIH). https://www.chj.es/es-es/medioambiente/SAIH/Paginas/Inicio.aspx; (iii) The Spanish National Meteorological Agency (AEMET). http://www.aemet.es/es/portada; (iv)The Atlas of Solar Radiation in Spain using climate data from Satellite Application Facilities (SAF EUMETSAT). https://www.aemet.es/documentos/es/serviciosclimaticos/datosclimatologicos/atlas_radiacion_solar/atlas_de_radiacion_24042012.pdf; (v) Data and cartography provided by JRB. Restrictions apply to the availability of these data. Data was obtained JRB and are available from the authors with the permission of JRB.

## References

[1] EPA (2005). EPA Guidelines for Responsible Pesticide Use.

[2] Azevedo A.S.O.N. (1998). Assessment and Simulation of Atrazine as Influenced by Drainage and Irrigation. An Interface between RZWQM and ArcView GIS. Ph.D. Thesis.

[3] Arias-Estévez M., López-Periago E., Martínez-Carballo E., Simal-Gándara J., Mejuto J.C., García Río L. (2008). The mobility and degradation of pesticides in soils and the pollution of groundwater resources. Agric. Ecosyst. Environ..

[4] CHJ (2015). Plan Hidrológico de la Demarcación Hidrográfica del Júcar, Memoria, Ciclo de Planificación Hidrológica 2015–2021.

[5] CHJ (2018). Estudios de Caracterización y Modelación de Procesos de Contaminación por Pesticidas en la Demarcación Hidrográfica del Júcar.

[6] Belenguer V., Martínez Capel F., Masiá A., Picó Y. (2014). Patterns of presence and concentration of pesticides in fish and waters of the Júcar River (Eastern Spain). J. Hazard. Mater..

[7] Ccanccapa A., Masiá A., Andreu V., Picó Y. (2016). Spatio-temporal patterns of pesticide residues in the Turia and Júcar Rivers (Spain). Sci. Total Environ..

[8] Masiá A., Camo J., Vázquez-Roig P., Blasco C., Picó Y. (2013). Screening of currently used pesticides in water, sediments and biotaof the Guadalquivir River Basin (Spain). J. Hazard. Mater..

[9] Fernández F., Marín J.M., Pozo O.J., Sancho J.V., Lòpez F.J., Morell I. (2008). Pesticide residues and transformation products in groundwater from a Spanish agricultural region on the Mediterranean Coast. Int. J. Environ. Anal. Chem..

[10] Menchen A., De Las Heras J., Gómez Alday J.J. (2017). Pesticide contamination in groundwater bodies in the Júcar River European Union Pilot Basin (SE Spain). Environ. Monit. Assess..

[11] Cabeza Y., Candela L., Ronen D., Teijon D. (2012). Monitoring the occurrence of emerging contaminants in treated wastewater and groundwater between 2008 and 2010. The Baix Llobregat (Barcelona, Spain). J. Hazard. Mater..

[12] Leonard R.A., Cheng H.H. (1990). Movement of pesticides into surface waters. Pesticides in the Soil Environment: Processes, Impacts, and Modeling.

[13] Ravi V., Johnson J.A. (1992). PESTAN: Pesticide Analytical Model Version 4.0 User’s Guide.

[14] Cheng Y., Zhou J.-Y., Shan Z.-J., Kong D. (2007). SCI-GROW Model for Groundwater Risk Assessment of Pesticides. J. Ecol. Rural. Environ..

[15] Voss C.I., Provost A.M. (2002). A Model for Saturated-Unsaturated Variable-Density Ground-Water Flow with Solute or Energy Transport.

[16] Šimůnek J., Genuchten M., Šejna M. (2018). The HYDRUS Software Package for Simulating the Two- and Three-Dimensional Movement of Water, Heat, and Multiple Solutes in Variably-Saturated Porous Media.

[17] Beltman W.H.J., Ter Horst M.M.S., Adriaanse P.I., De Jong A. (2006). Manual of FOCUS_TOXSWA Version 2.2.1.

[18] Van den Berg E., Tiktak A., Boesten J., Van der Linden T. (2016). PEARL Model for Pesticide Behaviour and Emissions in Soil-Plant Systems.

[19] Boesten J.J.T.I., Gottesbüren B. (2000). Testing PESTLA by two modellers for bentazone and ethoprophos in a sand soil. Agric. Water Manag..

[20] Scorza Júnior R.P., Boesten J.J.T.I. (2005). Simulation of pesticide leaching in a cracking clay soil with the PEARL model. Pest Manag. Sci..

[21] Di H.J., Anylmore L.A.G. (1997). Modeling the Probabilities of Groundwater Contamination by Pesticides. Soil Sci. Soc. Am. J..

[22] Teklu B.M., Adriaanse P.I., Ter Horst M.M., Deneer J.W., Van den Brink P.J. (2015). Surface water risk assessment of pesticide in Ethiopia. Sci. Total Environ..

[23] Huff Hartz K.E., Edwards T.M., Lydy M.J. (2017). Fate and transport of furrow-applied granular tefluthrin and seedcoated clothianidin insecticides: Comparison of field-scale observations and model estimates. Ecotoxicology.

[24] D’Andrea M.F., Letourneau G., Rousseau A.N., Brodeur J.C. (2020). Sensitivity analysis of the Pesticide in Water Calculator model for applications in the Pampa region of Argentina. Sci. Total Environ..

[25] Chen H., Zhang X., Demars C., Zhang M. (2017). Numerical simulation of agricultural sediment and pesticide runoff: RZWQM and PRZM comparison. Hydrol. Process..

[26] Young D.F., Fry M.M. (2020). PRZM5 A Model for Predicting Pesticides in Runoff, Erosion, and Leachate Revision B.

[27] Young D.F. (2016). Pesticide in Water Calculator User Manual for Versions 1.50 and 1.52.

[28] Young D.F., Goh K.S., Gan J., Young D.F., Luo Y. (2020). U.S. Environmental Protection Agency Model for Estimating Pesticides in Surface Water. Pesticides in Surface Water: Monitoring, Modeling, Risk Assessment, and Management.

[29] Rodrigo-Ilarri J., Rodrigo-Clavero M.-E., Cassiraga E., Ballesteros-Almonacid L. (2020). Assessment of Groundwater, contamination by Terbuthylazine using vadose zone numerical models. Case Study of Valencia Province (Spain). Int. J. Environ. Res. Public Health.

[30] Loague K., Lloyd D., Nguyen A., Davis S.N., Abrams R.H. (1998). A case study simulation of DBCP groundwater contamination in Fresno County, California 1. Leaching through the unsaturated subsurface. J. Contam. Hydrol..

[31] Trevisan M., Errera G., Goerlitz G., Remy B., Sweeney P. (2000). Modelling ethoprophos and bentazone fate in a sandy humic soil with primary pesticide fate model PRZM-2. Agric. Water Manag..

[32] Ma Q., Hook J.E., Wauchope R.D., Dowler C.C., Jhonson A.W., Davis J.G., Gascho G.J., Truman C.C., Sumner H.R., Chandler L.D. (2000). GLEAMS, Opus, PRZM2β, and PRZM3 Simulations Compared with Measured Atrazine Runoff. Soil Sci. Soc. Am. J..

[33] Richards L.A. (1931). Capillary conduction of liquids through porous mediums. Physics.

[34] Suárez L.A., U.S. Environmental Protection Agency, National Exposure Research Laboratory (2005). PRZM A Model for Predicting Pesticide and Nitrogen Fate in the Crop Root and Unsaturated Soil Zones: User’s Manual for Release 3.12.2.

[35] NRCS (1986). Urban Hydrology for Small Watersheds TR-55.

[36] Williams J.R. (1975). Sediment Yield Prediction with Universal Equation Using Runoff Energy Factor.

[37] Lewis K.A., Tzilivakis J., Warner D.J., Green A. (2016). An international database for pesticide risk assessments and management. Hum. Ecol. Risk Assess..

[38] PPDB: Pesticide Properties DataBase [Internet] Hertfordshire: University of Hertfordshire; 2007 [Updated 25 August 2020]. http://sitem.herts.ac.uk/aeru/ppdb/en/index.htm.

[39] PubChem [Internet] National Library of Medicine, National Center for Biotechnology Information, 8600 Rockville Pike, Bethesda, MD, 20894 USA: [Updated March 2019]. https://pubchem.ncbi.nlm.nih.gov/.

[40] Plants EU Pesticides Database [Internet] European Commission; [Updated 7 April 2016]. https://ec.europa.eu/food/plant/pesticides/eu-pesticides-db_en.

[41] CHJ (2021). Caracterización Básica de las Masas de Aguas Subterránea. Confederación Hidrográfica del Júcar.

[42] Royal Decree 1514/2009. BOE. Ministerio de Medio Ambiente, y Medio Rural y Marino; Madrid, Spain, 2009. https://www.boe.es/buscar/pdf/2009/BOE-A-2009-16772-consolidado.pdf.

[43] Gustafson D.I. (1991). Groundwater ubiquity score: A simple method for assessing pesticide leachability. Environ. Sci. Technol..

[44] Estadística Anual de Consumo de Productos Fitosanitarios y Estadística Quinquenal de Utilización de Productos Fitosanitarios en la Agricultura [Internet] Ministerio de Agricultura, Pesca y Alimentación. https://www.mapa.gob.es/es/estadistica/temas/estadisticas-agrarias/agricultura/estadisticas-medios-produccion/.

[45] Sancho Ávila J.M., Riesco Martín J., Jiménez Alonso C., Sánchez de Cos Escuin M.C., Montero Cadalso J., López Bartolomé M. (2012). Atlas de Radiación Solar en España Utilizando Datos del SAF de Clima de EUMETSAT.

[46] Meyer A.F. (1944). Evaporation from Lakes and Reservoirs.

